# Decreasing cost of public sector first-line ART services in India from 2007-2008 to 2015-2016

**DOI:** 10.1371/journal.pone.0206988

**Published:** 2018-11-12

**Authors:** G. Anil Kumar, Rakhi Dandona, Bharat B. Rewari, S. G. Prem Kumar, Sukarma Tanwar, Marielle C. Gagnier, Venkata S. Vishnumolakala, Lalit Dandona

**Affiliations:** 1 Public Health Foundation of India, New Delhi, India; 2 Institute for Health Metrics and Evaluation, University of Washington, Seattle, WA, United States of America; 3 National AIDS Control Organisation, Ministry of Health and Family Welfare, Government of India, New Delhi, India; 4 World Health Organization Country Office for India, New Delhi, India; NPMS-HHC CIC / LSH&TM, UNITED KINGDOM

## Abstract

**Introduction:**

India has scaled-up antiretroviral treatment (ART) in public sector facilities, but data to understand time trends of average cost of ART are limited.

**Materials and methods:**

Cost and output data were collected at all public sector ART centres in undivided Andhra Pradesh (high-HIV burden state) and Rajasthan (low-HIV burden state) in India from fiscal year 2007–2008 to 2012–2013. Average cost per patient for first-line ART, and its relation with scale of services, were assessed. Using data on scale of services, the average cost was estimated up to 2015–2016. Break-even point was estimated from average and marginal cost functions. Costs were adjusted to 2015 constant price.

**Results:**

The average cost per patient alive and on ART in 2015–2016 was US$162 in undivided Andhra Pradesh and US$186 in Rajasthan, which was 51.4% and 35.8% lower than in 2007–2008, respectively. Average ART drug cost declined by 27.2% during this period, and was 70.9% and 61.5% of the total ART cost in the two states in 2015–2016. The average cost other than ART drugs declined by 73.1% and 45.7%, with the number of patients served increasing 7 and 14.2 times, respectively. Average cost other than ART drugs had a significant negative relation with scale (R^2^ = 86.4%-82.8%, p<0.001). Break-even analysis suggested that 47.5% and 58.8% of the ART centres in undivided Andhra Pradesh and Rajasthan, respectively, were functioning below optimal scale in 2015–2016. The estimated total economic cost of first-line ART services provided in the public sector in India in fiscal year 2015–2016 was US$ 151 million; it would be US$ 216.1 million to provide this to all eligible persons in India.

**Conclusion:**

The average cost of providing first-line ART has declined in India, and further reduction is possible if the optimal scale of services is achieved. These findings can inform resource requirement for the ART programme in India.

## Introduction

The introduction of antiretroviral treatment (ART) for people living with HIV/AIDS has been credited with significant improvement in the quality of life and reduction in mortality in HIV high burden countries including India [1]. With an estimated 2.1 million people living with HIV in 2015–2016, India has the third highest HIV burden in the world, after South Africa and Nigeria [1, 2]. In order to increase provision of ART services, the government of India started a programme to provide first-line ART drugs purchased by the Government and provided at no cost to HIV patients in the year 2004 initially in high-HIV prevalence states, with the plan to subsequently expand this to other states [3]. ART services have been scaled up rapidly in India over the last decade with close to 900,000 patients receiving ART through public sector ART centres in the fiscal year 2015–2016. These ART centres are standalone facilities located in public medical colleges, district hospitals and other peripheral health facilities in India to provide ART services to patients, including counseling, testing, medications and opportunistic infections care. The recommendation for the patients on ART is to visit the ART centre once every month for routine examination and collection of ART medication.

Analysis of the trends of average cost per patient alive and on ART in India up to the recent time would help inform policy in estimating the resources need to provide ART services, but such data on a large-scale are not readily available. We assessed how average cost of first-line ART service provision in the public sector changed from fiscal year 2007–2008 to 2015–2016 in undivided Andhra Pradesh (a high-HIV burden state) and Rajasthan (a relatively low-HIV burden state) in India, and extrapolated this to estimate the resources needed to provide first-line ART service across India. The estimated adult HIV prevalence in Andhra Pradesh was 0.66% in 2015, and was 0.23% in Rajasthan [2]. Andhra Pradesh in south India had a population of about 85 million population in 2013 and the highest number of persons with HIV among any Indian state with a long-standing ART programme, and Rajasthan in north India had a population of about 70 million and a relatively lower HIV burden with a more recent ART programme [3].

## Materials and methods

This study was approved by the Health Ministry Steering Committee of the Indian Council for Medical Research and by the National AIDS Control Organisation (NACO). Ethics approval for this study was obtained from the Ethics Committees of Public Health Foundation of India, New Delhi and the University of Washington, Seattle, USA.

Details of the study design are described elsewhere [4]. A brief description follows. For this study, we sought cost data from all 44 ART centres in Andhra Pradesh and all 11 ART centres in Rajasthan that were functional for more than 6 months at the time of data collection in 2012–2013. Formal consent to collect data was obtained from the medical officer of the ART centres. Two ART centres in Andhra Pradesh did not provide data. Detailed data were initially collected from ART centres in Andhra Pradesh during February-May 2013 and from Rajasthan ART centres during June-July 2013. These data were supplemented in 2016 with ART programme data up to the fiscal year 2015–2016. Andhra Pradesh state was divided into two states, Andhra Pradesh and Telangana, in June 2014. As the initial field data collection for this study was done in 2013 prior to this split, we report the findings for undivided Andhra Pradesh.

### Cost and services data from undivided Andhra Pradesh and Rajasthan

Data on outputs including the number of patients alive and on-ART, patients ever enrolled on-ART and patients registered pre-ART, and detailed cost data were collected for the last five completed fiscal years at the time of data collection–from April 2007 to March 2012 for undivided Andhra Pradesh and from April 2008 to March 2013 for Rajasthan.

Health economic evaluation can be carried out from different perspectives. We considered the healthcare sector perspective economic cost to provide ART services in India. The economic cost of ART was estimated in five categories: personnel, capital goods, building rentals, recurring goods and recurring services costs [5–7]. The cost of managing opportunistic infections (OIs) related to HIV was estimated separately and added to each of these categories. The items considered for the computation of cost under each of these categories are shown in Box 1.

**Box 1. Items included in computing various cost components**

***Personnel–***Includes total remuneration (cash or in kind) paid to the employee of ART centre for ART and OI services during the reference period. Personnel cadres at ART centre include medical officer(s), staff nurse(s), counsellor(s), pharmacist, laboratory technician, data manager(s), and community care coordinator.

***Capital goods***–Includes tangible and durable assets (equipment and machinery) used at the ART centre to provide ART and OI services to patients. Items include furniture, electrical fixtures, air conditioner, refrigerator, computer, printer, needle and syringe destroyer, weighing machine, CD4 count machine, wheel chair, BP apparatus, etc.

***Building rental****–*Includes the estimated rental cost of a building used for the provision of ART services to patients.

***Recurring goods***–Includes ART drugs, drugs for OIs, medical consumables such as laboratory reagent, gloves, suture kits, test tubes, slides, syringes, laboratory tests such as CD4 count test, HIV test, OIs test, health records system, stationary, etc.

***Recurring services***–Includes training of staff, repair or renovations, electricity and water, internet connection and computer charges, waste disposal, photocopying, postage and courier, and facility administration related expenses.

Personnel costs included salary and benefits of staff contributing to the work of ART centres and to the management of OIs, which were extracted from the records at ART centres. If the staff contributed partially to these services, pro rata cost was computed. The cost of capital goods were collected from the asset registers at ART centres. If cost of any capital good was not available, its market price for the year of purchase was obtained. Assuming a 5-year life of the capital goods (equipment and machinery), one-fifth of the total cost was allocated to each fiscal year. Since all ART centres were part of public hospitals, there was no rent paid. We estimated the economic rental cost based on the floor area of each ART centre and the average rental rate for establishments in that area. The recurring goods cost included first-line ART and OI drugs, laboratory tests for ART and OIs, and other consumables. Most of these items are supplied to the ART centre by NACO through the State AIDS Control Society (SACS). The first-line generic ART drugs are procured by NACO through bids. These drugs are paid for by NACO and supplied directly to SACS, which distributes these to each ART centre in the state. These ART drugs are provided at no cost to the patients. The combinations of first-line ART drugs used in India during the study period were Stavudine-based regimen with/without Efavirenz and Zidovudine-based regimen with/without Efavirenz. The average cost of ART drugs per patient was obtained from NACO for each year. The cost of other reccuring goods was obtained from NACO, SACS and ART centres as relevent. For some items for which costs were not available, we estimated those from market prices. Under recurrent services, the cost for staff training was extracted from the financial budgets provided for training by SACS. The actual cost of most reccurent services were extracted from the records at ART centres, and if not available estimated from market prices. Since all ART centres are part of public hospitals, some of the items were shared for which we apportioned the cost for ART services. This was the best possible approach based on the data available. Data were entered directly in Datstat Illume Survey Manager 5.1 software (DatStat Inc, Seattle, WA).

The average cost per patients alive and on-ART of first-line ART services for each year was computed by dividing the estimated total economic cost for ART services for each facility in each state by the number of persons alive and on ART in a particular year. We provide ranges for the average overall cost per patient between the facilities in each state for every year, which conveys the variability of the average cost estimates. The details of how each item was costed in the various cost components in the two states are provided in S1 File.

### Relation between average cost and scale

We obtained the number of patients alive and on ART up to 2015–2016 for both the states from NACO. We defined scale as the number of patients alive and on-ART served by an ART clinic. As the ART drug cost was similar across ART centres in India at any given time due to common procurement through NACO, we considered the average cost other than ART drugs per patient as an indicator of efficiency. Using the cost and scale data for the last year for which detailed data had been collected, 2011–2012 for undivided Andhra Pradesh and 2012–2013 for Rajasthan, we explored the relation between scale and average cost other than ART drugs using regression fits separately for each state. We explored different types of bivariate regressions (exponential, linear, logarithmic, polynomial and power) to find the best fit. Using the best relation from these explorations, we estimated the average cost other than ART drugs up to 2015–2016 in the two states based on the scale of service in each year in the two states. We then added this for each year to the average ART drug cost obtained from NACO to estimate the average cost per patient alive and on ART. A retrospective time series analysis was carried out to assess the trends of the average cost per patient alive and on ART and of the ART drug component versus other costs.

### Break-even point calculation

We estimated the conventional total economic cost function by least squares regression, using patients alive and on-ART as a function of total cost for the year 2015–2016 and derived the average and marginal cost function, separately for each state. Using these two functions, we arrived at the break-even point for each state separately, which is the point of intersection of the average and marginal economic cost curves at which the economic cost of an extra unit of output is the same as the economic cost per unit output [8–10]. When the average and marginal economic cost curves intersect the economic cost of an extra unit of output is the same as the economic cost per unit output. We considered this point as the optimal scale to achieve efficiency. For all those facilities that were operating below this optimal scale, there would be scope to further increase demand (scale) for services that would yield increasing returns to scale, as the average cost of providing services are still falling and the marginal cost of providing service to one additional patient is less than the average cost. We calculated the proportion of ART centres with the number of patients alive and on-ART below the break-even point in 2015–2016. The details of these calculations are provided in S2 File.

### Extrapolation to India

We obtained data on the number of patients alive and on ART for the fiscal years 2007–2008 to 2015–2016 for all states and union territories in India, and on the cost of ART drugs for each year, from NACO. We divided the Indian states into four high-HIV burden south states (Andhra Pradesh, Karnataka, Maharashtra and Tamil Nadu) which made-up about 60 percent of the persons living with HIV in 2007–2008 as one group, and the other states as another group [2]. Using the number of patients alive and on ART in these two groups of states and the estimated average ART cost, we estimated the total ART cost in these two groups for the fiscal years 2007–2008 and 2015–2016, applying average ART cost in undivided Andhra Pradesh to the four high-HIV burden states and the average ART cost in Rajasthan to the other states. We estimated the average ART cost for India using the weighted average of these two groups. We applied this average ART cost for India to the total number of patients estimated to be eligible for ART in 2015–2016 by NACO inorder to obtain the total cost that would have been incurred if all eligible patients would have received ART.

### Cost at 2015 constant price

All costs presented in this report are in 2015 constant US dollars (US$). The cost in Indian Rupees for each fiscal year was first converted to the 2015 constant price using the gross domestic product deflator [11]. These INR figures were then converted to US$, using the average exchange rate of Indian Rupees 65.46 for the 2015 fiscal year [12].

## Results

The number of ART centres functioning for the compete fiscal year 2007–2008 was 24 and 2 in undivided Andhra Pradesh and Rajasthan, which increased to 61 and 17 in 2015–2016, respectively (Table 1). A total of 192,597 and 23,071 patients alive and on ART were served in undivided Andhra Pradesh and Rajasthan in 2015–2016, a 7 and 14.2 times increase from 2007–2008, respectively. The median number of patients alive and on ART across the ART centres in undivided Andhra Pradesh and Rajasthan was 3,417 and 1,075, respectively, in 2015–2016. The increase in this median number from 2007–2008 to 2015–2016 was much higher for undivided Andhra Pradesh than for Rajasthan.

**Table 1:** Patients alive and on ART in public sector ART centres in undivided Andhra Pradesh and Rajasthan states in India from the fiscal year 2007–2008 to 2015–2016.

Fiscal year	Undivided Andhra Pradesh			Rajasthan		
	No. of ART centres	Total number of patients alive and on ART	Mean number of patients alive and on ART (Range across ART centres)	No. of ART centres	Total number of patients alive and on ART	Mean number of patients alive and on ART (Range across ART centres)
2007–2008	24	27,481	1,145 (54–2,690)	2	1,629	815 (513–1,116)
2008–2009	25	44,740	1,790 (55–4,240)	5	4,749	950 (114–2,048)
2009–2010	31	66,024	2,130 (204–4,996)	6	7,097	1,183 (80–2,812)
2010–2011	39	88,219	2,262 (13–5,407)	7	9,445	1,349 (128–3,063)
2011–2012	42	113,251	2,696 (449–7,248)	10	12,292	1,229 (232–3,375)
2012–2013	46	125,651	2,732 (465–7,079)	11	15,747	1,403 (284–3,694)
2013–2014	51	159,518	3,128 (591–8,774)	16	18,299	1,144 (470–3,250)
2014–2015	54	176,821	3,274 (426–9,339)	17	21,729	1,278 (358–3,564)
2015–2016	61	192,597	3,157 (172–10,466)	17	23,071	1,357 (453–3,890)

The total economic cost of ART services and distribution of cost components of 42 ART centres in undivided Andhra Pradesh and 11 ART centres in Rajasthan for five years are shown in Table 2. ART drugs and other recurrent goods made-up the predominant proportion of the cost in both states in these five years. ART drugs were the single largest contributor to the total cost, 63.1% (range 44.1% - 73.6% across facilities) in undivided Andhra Pradesh and 56.3% (range 35.4% - 64.6%) in Rajasthan in the last year of data collection in each state. The average cost of diagnosing and manging opportunistic infections was 6.6% and 6.4% of the total cost in Andhra Pradesh and Rajasthan, respectively.

**Table 2:** Economic cost to public sector ART centres in undivided Andhra Pradesh and Rajasthan states in India from the fiscal years 2007–2008 to 2012–2013^a^.

Fiscal year ^a^	Undivided Andhra Pradesh						Rajasthan					
	Total economic cost in millions of US$^b^ (number of ART centres)	Percent of total economic cost (percent range across ART centres)					Total economic cost in millions of US$^b^ (number of ART centres)	Percent of total economic cost (percent range across ART centres)				
		Personnel	ART drugs	Recurring goods other than ART drugs	Recurring services	Other		Personnel	ART drugs	Recurring goods other than ART drugs	Recurring services	Other
2007–2008	9.47 (24)	5.0 (2.2–22.4)	48.1 (56.5–60.3)	42.7 (22.1–52.3)	2.7 (0.2–20.4)	1.5 (0.2–13.3)						
2008–2009	11.73 (25)	5.9 (3.2–40.5)	54.9 (17.8–63.3)	34.7 (13.0–43.7)	3.1 (0.2–17.8)	1.5 (0.5–10.9)	1.29 (5)	8.4 (6.4–18.8)	53.9 (44.3–57.7)	30.3 (22.4–33.4)	5.2 (3.2–14.4)	2.3 (0.8–5.2)
2009–2010	14.64 (31)	6.7 (3.9–31.7)	57.9 (27.9–69.1)	30.9 (20.0–44.5)	3.0 (1.6–10.8)	1.5 (0.5–7.5)	1.78 (6)	9.8 (5.7–25.1)	52.7 (32.5–57.2)	29.0 (19.9–32.4)	6.5 (3.5–16.8)	2.1 (1.2–7.1)
2010–2011	18.50 (39)	7.7 (4.6–37.8)	58.4 (16.3–68.4)	29.4 (5.9–41.9)	3.0 (1.7–18.8)	1.5 (0.7–21.1)	2.22 (7)	11.0 (8.0–28.1)	53.5 (36.7–59.2)	28.0 (15.4–31.4)	5.1 (3.3–11.1)	2.5 (1.4–8.1)
2011–2012	20.90 (42)	8.3 (0.8–27.2)	63.1 (44.1–73.6)	24.3 (17.3–30.8)	2.8 (1.5–7.3)	1.5 (0.6–6.6)	2.79 (10)	13.7 (7.3–40.3)	52.9 (31.5–63.2)	25.4 (15.0–31.3)	5.3 (3.2–10.9)	2.7 (1.5–5.6)
2012–2013							2.90 (11)	14.0 (6.6–37.2)	56.3 (35.4–64.6)	22.4 (13.9–27.4)	4.7 (3.2–9.2)	2.7 (1.5–5.1)

^a^Data were collected for five years in each state, 2007–2008 to 2011–2012 in undivided Andhra Pradesh and 2008–2009 to 2012–2013 in Rajasthan

^b^All costs are in 2015 constant price. US$ 1 = Indian Rupees 65.46 (average exchange rate in the year 2015)

**Fig 1:**
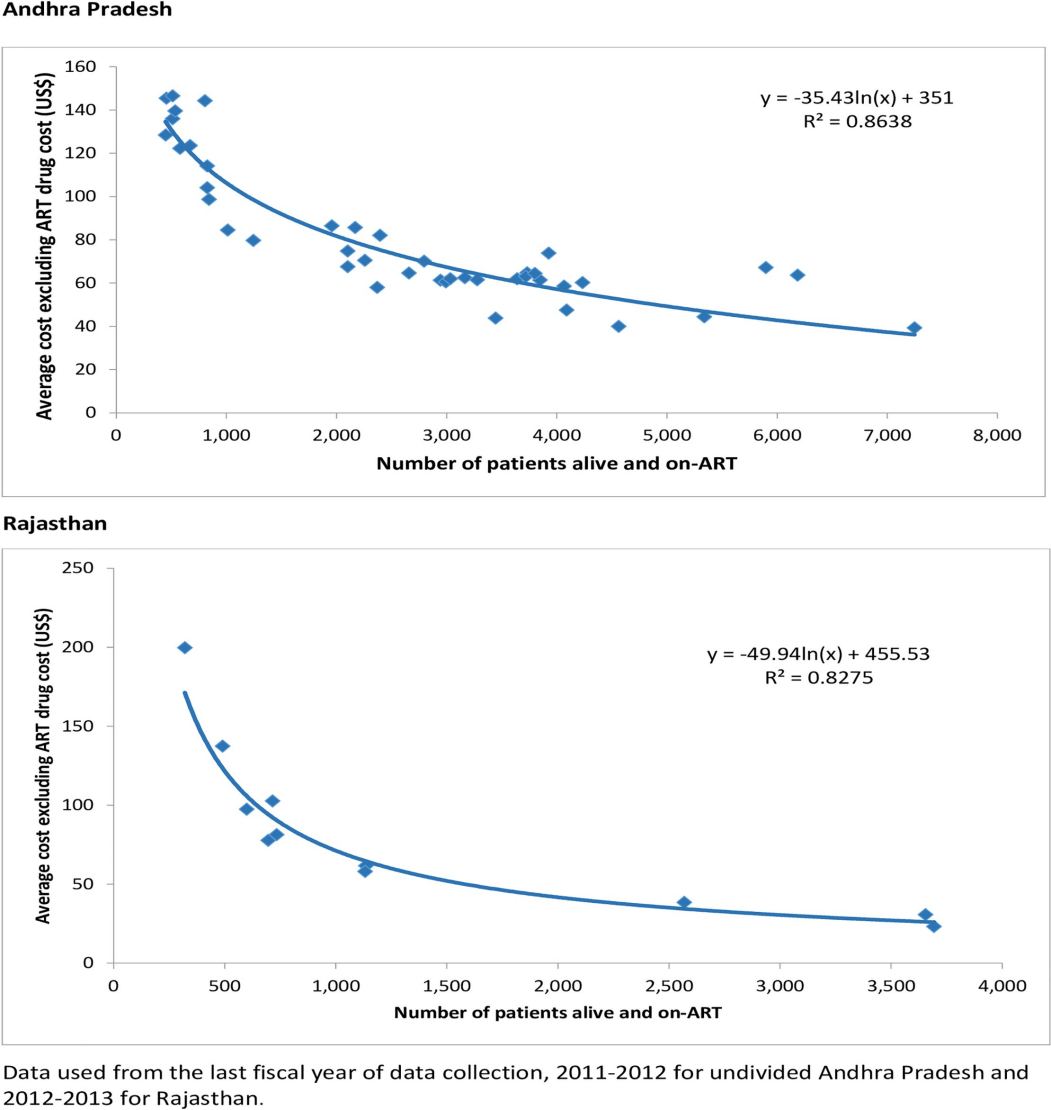
Relation between scale and other than ART drug cost per patient on ART for ART centres in undivided Andhra Pradesh and Rajasthan states in India.

The average cost other than ART drugs per patient had significant negative relation with scale. The best fit for this relation was obtained with the logarithmic function: R^2^ = 86.4% for undivided Andhra Pradesh and R^2^ = 82.8% for Rajasthan, p<0.001 (Fig 1).

Table 3 shows the trends of average cost per patient alive and on ART, the average cost of ART drugs and average cost other than ART drugs, for undivided Andhra Pradesh and Rajasthan from 2007–2008 to 2015–2016. The overall average cost per patient alive and on ART dropped during this period by 51.4% from US$ 333 to US$ 162 in undivided Andhra Pradesh, and by 35.8% from US$ 290 to US$ 186 in Rajasthan.

**Table 3:** Average cost per patient alive and on ART in public sector ART centres in undivided Andhra Pradesh and Rajasthan states in India from fiscal year 2007–2008 to 2015–2016.

Year	Undivided Andhra Pradesh					Rajasthan				
	No. of ART centres	Average cost (US$)^a^ ^b^			Other cost as percent of overall average cost	No. of ART centres	Average Cost (US$)^a^ ^b^			Other cost as percent of overall average cost
		Overall cost (range across ART centres)	ART drugs cost	Other cost (range across ART centres)			Overall cost (range across ART centres)	ART drugs cost	Other cost (range across ART centres)	
2007–2008	24	333 (320–589)	158	175 (162–431)	52.5	2	290 (287–337)	158	132 (129–179)	45.6
2008–2009	25	267 (265–546)	145	121 (119–411)	45.5	5	274 (254–302)	145	128 (108–157)	46.9
2009–2010	31	220 (204–466)	127	93 (78–340)	42.4	6	243 (222–370)	127	117 (96–243)	48.0
2010–2011	39	183 (179–343)	106	77 (73–237)	42.0	7	201 (186–324)	106	95 (80–218)	47.1
2011–2012	42	161 (152–220)	101	60 (51–119)	37.3	10	194 (171–250)	101	93 (70–149)	47.9
2012–2013	46	153 (128–217)	94	59 (34–124)	38.8	11	168 (146–284)	94	75 (52–190)	44.3
2013–2014	51	140 (114–197)	88	52(26–109)	36.9	16	171 (133–218)	88	83 (45–129)	48.3
2014–2015	54	134 (109–201)	85	48 (24–115)	36.0	17	161 (125–222)	85	75 (40–137)	46.9
2015–2016	61	162 (135–255)	115	47 (21–140)	29.1	17	186 (150–239)	115	72 (36–125)	38.5

^a^Average costs were estimated for undivided Andhra Pradesh for 2012–2013 to 2015–2016, and for Rajasthan for 2007–2008 and 2013–2014 to 2015–2016, using the relation of average cost with scale at ART centres in 2011–2012 and 2012–2013 as explained in the methods section

^b^All costs are in 2015 constant prices. US$ 1 = Indian Rupees 65.46 (average exchange rate in the year 2015)

The average cost for ART drugs declined by 27.5% from US$ 158 in to US$ 115 in 2015–2016. During this period, the average cost component other than ART drugs declined by 73.1% for undivided Andhra Pradesh from US$ 175 to US$ 47, and 45.7% for Rajasthan from US$ 132 to US$ 72.

**Fig 2:**
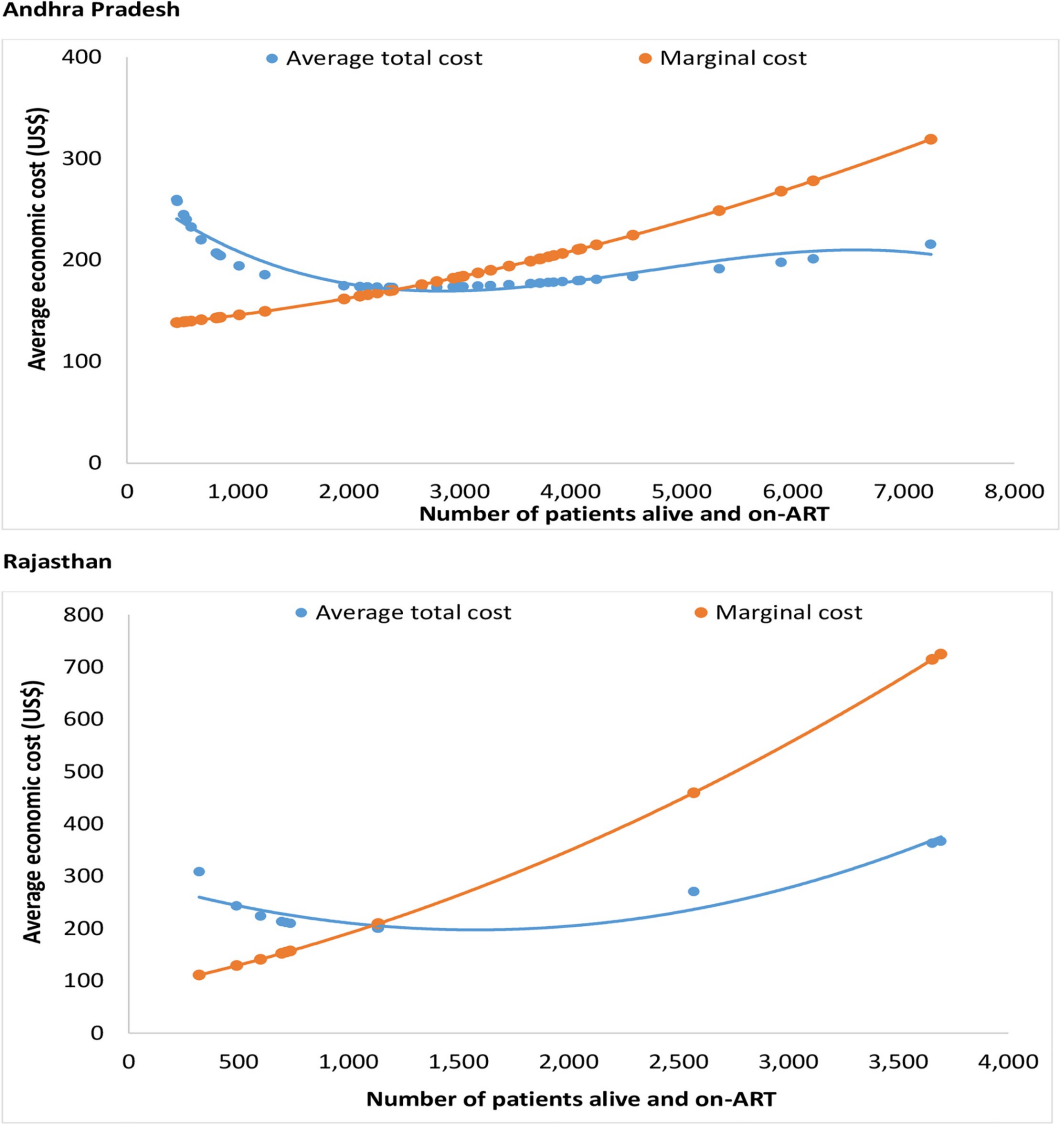
Break-even point for ART centres in Andhra Pradesh and Rajastan 2015–2016.

The break-even point for the number of patients alive and on ART was 2,735 for undivided Andhra Pradesh and 1,259 for Rajasthan in 2015–2016 (Fig 2). At any point above this level the marginal economic cost of providing treatment for an extra patient is less than the average economic cost. In 2015–2016, 47.5% and 58.8% of the ART centres were operating below the break-even point in undivided Andhra Pradesh and Rajasthan, respectively.

Table 4 shows the estimated total cost of first-line ART provision in public sector ART centres in two groups of states in India in 2007–2008 and 2015–2016. Between 2007–2008 and 2015–2016, the number of patients alive and on ART increased 6.1 and 8.3 times, and the total cost of first-line ART provision increased 3.1 and 5.4 times, respectively, in the four high-burden and other states of India. ART drugs made up 67.6% of the total ART service provision cost in India in 2015–2016.

**Table 4:** Estimated cost of first-line ART in public sector ART centres in the four high-HIV burden states and the other states in India in fiscal years 2007–2008 and 2015–2016.

Year		Population (millions)		Total number of patients alive and on ART	Total cost of ART services (US$, millions)^b^	ART drugs cost (US$, millions)^b^	ART drugs cost as percent of total cost
**2007–2008**	Four high- HIV burden states^a^		315	97,182	32.4	15.4	47.4
	Other states		817	35,877	10.4	5.7	54.5
	India		1,132	133,059	42.8	21.0	49.2
**2015–2016**	Four high- HIV burden states^a^		350	590,216	95.6	67.9	71.0
	Other states		964	297,611	55.4	34.2	61.8
	India		1,314	887,827	151.0	102.1	67.6

^a^The four high-burden south states are Andhra Pradesh, Karnataka, Maharashtra and Tamil Nadu

^b^All costs are in 2015constant prices. US$ 1 = Indian Rupees 65.46 (average exchange rate in the year 2015)

USD$ 151 million was estimated to have been spent on providing first-line ART services in India in 2015–2016. If all of the 1.27 million person estimated by NACO to be eligible for ART in 2015–2016 had received these services [2], the total cost would have been USD$ 216.1 million.

## Discussion

The average cost to provide ART services per patient has declined substantially in India over the past decade or so. Comparing costs at the 2015 constant prices, there was a 27% decrease in the cost of ART drugs from 2007–2008, and a more impressive 69% decrease in other costs in the high-HIV burden undivided Andhra Pradesh state and a 44% decrease in other costs in the relatively low-HIV burden Rajasthan state due to increasing scale of services in the ART centres in these two states. This reduction in cost other than ART drugs indicates an improvement in efficiency of service provision, which was relatively more efficient in Andhra Pradesh due to a longer standing public funded ART programme compared with Rajasthan. The break-even point for the ART centres in these states suggested that in 2015–2016 there was scope to further improve efficiency by increasing scale of services at about half the public sector ART facilities in undivided Andhra Pradesh and about 60% of the facilities in Rajasthan. There were wide variations in the disaggregated cost categories between the ART centres in each state, which could be contributing to the relatively lower efficiency in some centres. It is useful to note here that attempts at demand creation to increase scale would incur cost. On the other hand, higher ART services would have the benefit of reducing HIV transmission, which would ultimately lower the ART requirement.

Applying the average cost per patient on ART, the total cost of first-line ART service provision at the public sector ART centres in India was US$ 151 million of which 68% was for ART drugs. This cost is only for first-line ART service provision, and does not include other ART programme costs or the cost of second- and third-line ART drugs. The first-line drug regimen was changed in India in 2015–2016, which led to a higher ART drug cost than in the preceding years. The second-line ART drugs were started in India’s ART programme in 2008–2009. The cost of these drugs is about twice that of the first-line ART drugs. It is estimated that about 2 percent of the adults on ART in India were on second-line ART in 2015–2016, approximately 16,000–17,000 patients. Third-line ART treatment was started in the programme in India in 2015–2016, and the coverage of this quite minimal so far. The cost for third-line ART drugs is about 16 times that of the first-line ART drugs.

The number of patients alive and on ART in the public sector facilities in India was reported to be about 888,000 in the year 2015–2016, an increase by 6.7 fold from the year 2007–2008. For providing first-line ART services to these patients in 2015–2016, cost of US$ 151 million was incurred in India. If all of the 1.27 million persons estimated by NACO to be eligible for ART in 2015–2016 had received these services, the total resourse requirement would have been USD$ 216 million. The current average ART cost estimates in this report, average US$170 for India, and its decreasing trend with increasing scale, can be useful for the ART programme to estimate resource requirement for ART service provision as India scales up these services further. Estimates of the cost of second- and third-line ART treatment and other programme costs could be added for a complete estimate of resource requirement for each state and for India as a whole.

A previous costing study of seven ART centres in India reported an ART average cost of US$ 353 in 2004–2006 [13], which we estimate to be US$ 413 at 2015 constant price. Data from another study of a non-governmental ART centre in south India from 2003–2005 suggested an ART average cost of US$ 557 at 2015 constant prices [14]. While these studies are not directly comparable with ours due to some differences in methods, their data generally support a declining trend in ART average cost in India when seen in conjunction with our data. Previous studies reporting ART average cost from sub-Saharan African countries for data years 2007 onwards show a wide variation [15–23], and the comparison with the ART average cost in India in our study suggests that at the 2015 constant prices the unit cost in the sub-Saharan African countries is generally higher than in India. A recent analysis has reported that there is significant scope for increasing the number of patients served at ART facilities in Kenya, Uganda and Zambia and improving the efficiency of these facilities [24].

There are limitations in our estimation of average cost that have to be considered while interpreting our findings. First, for calculating the average ART cost, we considered only alive patients as denominator due to unavailability of complete data on patients lost to follow-up, which could have biased the estimated average cost per patient on ART. However, only 8.8% patients were lost to follow-up in this study [4], thus the extent of this potential bias is unlikely to be large. Second, this study considered only the first-line ART costs, but since this is the major component of the ART programme in India these findings are quite relevant. Finally, for the extrapolation to total cost for ART services in India, we considered the average cost in undivided Andhra Pradesh to estimate the cost of the four high- HIV burden states, and the average cost in Rajasthan for all other low burden states. This extrapolation may not be exactly applicable to the other states, but because ART drug cost is the major component, which is the same for all states as the drugs are procured centrally through NACO, this extrapolation seems reasonable. Despite these limitations, this study provides the most recent and comprehensive evidence base for the cost of ART services per adult patient in India over the past 10 years.

The declining average cost per ART patient over the past decade in both high- and low-HIV burden states in India, and the lower unit cost as compared with sub-Saharan countries, are encouraging. Our findings suggest that further improvement in efficiency and reduction of average cost are possible by increasing scale of services in facilities where it is currently low. The ART programme in India could pay particular attention to this aspect to maximize efficiency and impact. It is possible that attempts to increase the patient volume could adversely impact the quality of care if attention is not given to maintaining quality of services. As the volume of patients increase further, the programme should have adequate training mechanism to maintain the required skills for all the ART staffs and commensurate with the enhancement of infrastructure as well required to ensure the quality of care. The findings in this report can form a valuable basis for costing of the ART services across the different states in India as the ART programme expands further.

## Supporting information

S1 File Costing details of various cost components for Andhra Pradesh and Rajasthan. (XLSX)

S2 File Break-even point analysis. (DOCX)
