# Supplementary material for: Decreasing cost of public sector first-line ART services in India from 2007-2008 to 2015-2016
Source: PLoS One. 2018 Nov 12;13(11):e0206988. doi: 10.1371/journal.pone.0206988 (PMC6231637; doi:10.1371/journal.pone.0206988)
Supplement: S2 File — (DOCX) [file pone.0206988.s002.docx]

**Decreasing cost of public sector first-line ART services in India from 2007-2008 to 2015-2016**

**Break-even point analysis**

The break-even point analysis is a method used in this analysis to determine that how many persons should be served at each ART for maximum efficiency of resource utilization. The efficiency is maximum at the break-even point.

Using the total cost and patients alive on ART in each ART centres, we derived a conventional total economic cost function by least squares regression. This cost function was a cubic equation; f(x) = C + b_1_ X + b_2_X^2^ + b_3_X^3^, where f(x) is the cost function for total economic cost, b_1_, b_2_, b_3_ are the derived coefficients, X is the total patients alive and on ART in each ART centres and C is the constant. Accordingly, we estimated the average and marginal cost for all the ART centres in each states separately for the last financial year considered for data collection using the cost function.

Total economic cost divided by that level of output gives average economic cost per unit output. The cost of generating one extra unit of output is called the marginal economic cost. The slope of the total economic cost function at various levels of output gives the marginal economic cost at that level of output. The locus of the various marginal economic cost points gives the marginal economic cost curve. The slope is calculated with the help of differential calculus, using the first order derivative. The break-even point was arrived at by solving the average and marginal cost functions for the last financial year. The point of intersection of the average economic cost and the marginal economic cost curves was the break-even point at which the economic cost of an extra unit of output was the same as the economic cost per unit output. This point was arrived at by equating the average economic cost and the marginal economic cost functions and solving for number of patients alive and on-ART [1,2].

For this analysis, we derived the average and marginal cost functions for each state separately using the cost function as the first and second derivate of the cost function. Using these functions, we estimated average and marginal cost for all the ART centres in each states separately for the financial year financial year 2015 and arrived the break-even point, which is the point of intersection of the average and marginal economic cost curves at which the economic cost of an extra unit of output is the same as the economic cost per unit output. Then we calculated the proportion of ART centres with number of patients alive and on-ART below the break-even point.

**References**

1. Samuelson PA, Nordhaus WD. Economic analysis of costs. Economics. New Delhi: Tata McGraw-Hill; 2001. p. 125-41.

2. Henderson JM, Quandt RE. The theory of the firm. Micro-Economic Theory: A Mathematical Approach. Tokyo: McGraw-Hill Kogakusha; 1971. p. 52-102.
